# School funding and educational attainment in adolescence: a longitudinal ecological study of English local authorities from 2015 to 2023

**DOI:** 10.1136/jech-2025-224132

**Published:** 2026-06-11

**Authors:** Lateef Akanni, Michelle Black, Davara Bennett, Yu Wei Chua, Hanna Creese, Obioha C Ukoumunne, G J Melendez-Torres, Dougal Hargreaves, Benjamin Barr, David Taylor-Robinson

**Affiliations:** 1 Department of Urban Studies and Social Policy, University of Glasgow, Glasgow, UK; 2 Department of Public Health, Policy and Systems, University of Liverpool, Liverpool, UK; 3 Department of Primary Care and Public Health, Imperial College London, London, UK; 4 University of Exeter, Exeter, UK

**Keywords:** EDUCATION, ADOLESCENT, GEOGRAPHY, POLICY, PUBLIC HEALTH

## Abstract

**Background:**

Adequate education funding is important to reduce the socioeconomic inequality in educational attainment, an important social determinant of health. Over the past decade, there have been reductions in public funding for schools in England, despite the introduction of a national funding formula (NFF) policy, aimed at reducing historical differences in school funding. We assessed the impact of school spending cuts on student attainment at age 16, additionally by deprivation and pre-NFF and post-NFF policy implementation.

**Methods:**

Our longitudinal ecological study covers the period 2014/2015 to 2022/2023, analysed at local authority (LA) level. Our exposure was secondary school spending per pupil using school-level data, aggregated by LA and inflation adjusted. Our outcome was General Certificate of Secondary Education (GCSE) attainment using average GCSE Attainment 8 scores across LAs. We used correlated random-effects regression to quantify the association between change in spending and change in GCSE attainment. We additionally analysed the association between spending cuts and attainment by LA deprivation, and before and after the NFF policy.

**Findings:**

Between 2015 and 2023, per pupil school spending decreased by 13% from £6367 to £5556, while average GCSE attainment declined by 2.1 points from 48.5 to 46.4. A 10% spending cut was associated with a 0.42-point decrease in average attainment (95% CI 0.19 to 0.66). The estimates are different across LA deprivation quintile-based groups. Most deprived LAs had a greater impact (0.54; 95% CI 0.17 to 0.92), compared with least deprived LAs (−0.04; 95% CI −0.46 to 0.38). The association after the NFF policy was not different from the pre-NFF period (p=0.639).

**Conclusion:**

School spending cuts were associated with decreased GCSE attainment across England, with a bigger impact in socioeconomically disadvantaged areas. Against a backdrop of spending cuts, NFF introduction did not narrow inequities in attainment. More equitable school investment is critical to improving attainment and reducing inequalities across the life course.

**WHAT IS ALREADY KNOWN ON THIS TOPIC**

**WHAT THIS STUDY ADDS**

- Increased adverse trends in adolescence educational attainment over time are partly explained by cuts to education spending.
- The impact of spending cuts was more pronounced in deprived geographical areas.
- Policy reform to bridge the historical funding gap did not reduce the pre-existing inequities in spending cuts and declining attainment.

**HOW THIS STUDY MIGHT AFFECT RESEARCH, PRACTICE OR POLICY**

- Increased school funding with equitable allocation and distribution formula may help improve educational attainment and reduce the disadvantage gaps.
- Adequate investment, together with an understanding of contextual and enabling school factors, will impact student experience, well-being and attainment.

## Introduction

 Education is a major determinant of health, and adequate education funding is an important consideration to reducing socioeconomic inequality in educational attainment, especially for those with greatest need.^1 2^ However, cuts to school funding in England could plausibly have contributed to the declining student performance and rising inequality in student outcomes.^3^ Between 2019 and 2023, the disadvantage gap index at the end of final secondary school examinations, typically taken at age 16 in England, widened from 3.7 to 3.9.^4^ The disadvantage gap index measures the attainment gap in General Certificate of Secondary Education (GCSE) examination results between pupils from disadvantaged and non-disadvantaged backgrounds in English schools.

Equitable funding for schools is an important policy objective for education, health and economic reasons. Linking better outcomes to education funding strengthens the need for more investment in education.^5^ More educated people, on average, have better employment and economic outcomes, including higher earnings, greater job satisfaction and reduced mortality risks.^6^ Also, breaking down barriers to opportunity is high on the new England government agenda, and educational attainment at the end of secondary school is an important metric for evaluating progress towards this goal.^7^ A detailed description of the structure of education and school funding system in England is provided in online supplemental text box 1.

International empirical evidence has affirmed the importance of school spending on educational attainment. Jackson and Mackevicius^8^ did a meta-analysis of the impacts of public school spending, comprising early years through secondary schools, on student outcomes. They found a positive impact of policies increasing school spending on students’ test scores. However, few school-level analyses in England found a positive effect of school spending on outcomes.^9–11^ Between 2003 and 2007, characterised as a period of rapid increase in real-term school expenditure in England, Pugh *et al*^9^ found larger effects of spending for non-specialist schools than specialist schools, and for schools with above average percentage of pupils eligible for free school meals. Using data extended to 2010, Nicoletti and Rabe^10^ found that increased expenditure—especially on education support staff and learning resources—benefited pupils with special educational needs and helped close attainment gaps. No study using recent data in England, especially since austerity-related public spending cuts, has assessed the impact of spending on student outcomes, and their implications on geographical and socioeconomic inequalities in education outcomes.

The pathways linking education funding and examination outcomes have also been discussed in the literature. Most analyses use the educational production function (EPF) approach, linking school inputs, including funding, to student outcomes, while accounting for individual, peer, family and community factors.^12^ Baker and Knight^13^ identified two main pathways between spending and student academic achievement, comprising capital investment and current operational spending. While the former involves spending on facility improvements and the quality of indoor and outdoor spaces for student learning, the latter constitutes spending on personnel salaries, teaching and instruction materials and other daily operational costs.^13^ In addition to the two main spending blocks, intermediate pathways between spending and examination outcomes include student health, study quality, academic engagement and attendance, all of which are connected to and have been evaluated using the EPF framework.^14 15^

The impact of reduced funding on academic attainment could be greater for schools that are underfunded, and those that serve most students from disadvantaged backgrounds, largely due to additional learning challenges experienced by students in poverty and minority groups.^5 16^ Indeed, Gorard and Siddiqui^17^ identified poverty and special educational needs as key predictors of poor performance. We also know from literature that there is a strong link between area-level deprivation and educational outcomes, with students from the most deprived areas facing greater challenges.^18^ The 2023 report of inspections and outcomes of state-funded schools in England by the Office for Standards in Education, Children’s Services and Skills shows a higher proportion of schools rated as inadequate and requiring improvement are in more deprived areas, with the gaps in education quality between least and more deprived higher among secondary than primary schools.^19^

We summarised the timeline of major reforms in mainstream school funding in England in online supplemental figure S1. The latest among these reforms was the national funding formula (NFF) introduced in 2016 to ensure fair and equitable distribution of school funding allocations based on specific needs and pupil characteristics, and to provide schools with budget stability.^20^ NFF primarily sought to redistribute existing resources more equitably across schools; it also introduced more rigid funding rules and reduced local authority (LA) discretion. The direct link between the NFF policy and student performance is complex and difficult to fully evaluate, as school funding is just one of many factors influencing student outcomes.^21^ However, structural changes to schools funding could alter how flexibly schools respond to funding changes, with implications for student outcomes.

In this study, our aim is to investigate the association between secondary school spending and academic attainment at age 16 across LAs in England over a period of austerity. Additionally, we evaluate whether the association varied by area-level deprivation, as less is known about how relative spending levels between more and less deprived LAs shaped the gaps in educational attainment. This is especially important considering the current government mission to improve social mobility and tackle child poverty through investment in education to expand opportunity.^7^ We also considered whether the association between school-level funding and pupil outcomes shifted after the introduction of the NFF—a change that, if present, may be indicative of structural shifts in how resources translate into outcomes under the new funding formula.

## Methods

### Study design

We performed a longitudinal ecological analysis using publicly available data between 2014/2015 and 2022/2023 academic years. We used 2015 and 2023, respectively, to refer to the start and end periods of our analysis.^22^ We used the 2019 local government boundaries corresponding to 151 upper tier LAs.^23^ LAs in England referred to geographical areas governing and responsible for providing various public services, including social care, waste collection, transportation and education.^24^ Our analysis included 146 LAs in England. Five LAs were excluded: three due to boundary changes and data inconsistencies during the period under consideration (Bournemouth, Christchurch and Poole; Cumbria; and Northamptonshire). The City of London was excluded as there are no state-funded secondary schools in the LA, while the Isles of Scilly were excluded because they have only one state-funded secondary school with data suppression due to low number of pupils.^25^ Hence, it is usually an extreme outlier and frequently excluded from ecological studies in England.^26 27^

### Data sources and variable measurement

Our outcome variable was attainment at the end of secondary school for pupils attending publicly funded secondary schools in England. Secondary schools funded through public grants in England include academies and LA-maintained schools. We measured educational attainment using the GCSE Attainment 8 score per pupil at LA level—a measure of the mean of pupils’ performance in eight GCSE-level qualifications at the end of secondary education. The Department for Education announced the Attainment 8 metric in 2013, with implementation commencing in 2016, as part of the England secondary schools’ accountability systems to report students’ results at the end of GCSE.^28^ Each GCSE subject was graded on a grading structure from 9 (highest) to 1 (lowest), corresponding to A-star to G. Attainment 8 was calculated by adding each student’s points across their eight best-performing subjects, which were divided into three buckets: the English and Mathematics; three English Baccalaureate subjects; and three other GCSE subjects.^29^ Both English and Mathematics were double weighted; however, when a student had taken both English Language and English Literature, only Mathematics was double weighted. The maximum Attainment 8 score was 90, indicating a 9-point score in all the eight GCSE subjects. GCSE data were sourced from the UK government education statistics database.^25^

Our main exposure variable was the annual school spending per pupil by all publicly funded secondary schools in each LA. We used individual school spending data for all state-funded secondary schools in England sourced from the Schools Financial Benchmarking database.^30^ All school spending data were mapped to upper tier LAs using the school postcode information and the Office for National Statistics (ONS) postcode to output area hierarchy lookup in England.^31^ We adjusted for annual inflation using the ONS Consumer Price Index (CPI). We used the mean monthly CPI from September to August, conforming to the academic year, indexed to the 2015 constant values.

We included as covariates time-varying potential confounders that change over time and that are likely to correlate with unobserved pupil efforts and pupils’ socioeconomic background influencing both the level of spending and educational attainment.^9 32^ The covariates include the number of full-time equivalent teachers, proportion of pupils eligible for free school meals, proportion whose first language is not English and proportion with special educational needs.

We examined the association between school spending and GCSE attainment by LA deprivation. Each LA in England was assigned to their within-country deprivation quintiles using the 2019 English Index of Multiple Deprivation.^23^ We also analysed the difference in the association before and after the NFF policy introduction using an NFF dummy variable, which equals 0 for periods before NFF (2015–2018) and equals 1 for periods after the NFF (2019–2023).

### Statistical analysis

First, we graphically explored trends in per pupil school spending and GCSE Attainment 8 scores in England. Second, we used scatter plots to visualise the association between the average absolute changes in school spending per pupil and GCSE performance, between 2015 and 2023. The scatter plots provide a visual assessment of the association and the distribution of mapped changes in exposure and outcome.

For the main analysis, we used correlated random-effects (CRE) regression model to estimate the association between changes in school spending and changes in GCSE attainment across all LAs (see the online supplemental appendix file 1). The strength of the CRE estimator, also referred to as Mundlak equivalence, is that it allows reduction in omitted variable biases by controlling for time-invariant unobserved heterogeneity that varies across LAs, while estimating time-invariant regressors in the estimation.^33^ We took the natural log of the real spending and divided the estimated coefficient by 10. We report the results as a change in average GCSE attainment associated with a 10% cut in average real-term spending over the study period. We adjusted for number of teachers, proportion of pupils eligible for free school meals, English as additional language and special educational needs.

We included an interaction term between school spending and LA deprivation to evaluate the differences in the association between school spending and GCSE attainment for each deprivation quintile. We also evaluated potential NFF policy effects by interacting school spending with the NFF indicator. We estimated the marginal effects of spending on GCSE attainment for each quintile of LA deprivation, and separately for the pre-NFF and post-NFF periods. We used the Wald test to assess the difference in effect modification between the pre-NFF and post-NFF period estimates.

We estimated all models using robust SEs clustered at LA levels to control for heteroskedasticity and account for correlations within groups. We also included period fixed effects to control for unobservable time-specific trends. All models are estimated using the ‘*xtreg*’ command, with the ‘*cre*’ option in StataNow. We evaluate the appropriateness of the estimated ‘*cre*’ model against a random-effects model using Mundlak specification test. Unlike the Hausman test, the Mundlak test allows for the evaluation of models with robust SEs in their estimation. We performed a sensitivity analysis of the estimated results to the COVID pandemic periods which impacted both school funding and GCSE outcomes. Schools received additional funding during the pandemic to cushion the impact of coronavirus. Also, there were changes in how GCSEs were assessed across England during these periods.^34^ We re-estimated models using data between 2015 and 2019. We also performed a robustness check of our findings to confounder adjustments and excluded the NFF interaction to allow for year-specific and not period fixed effects.

## Results

**Figure 1:**
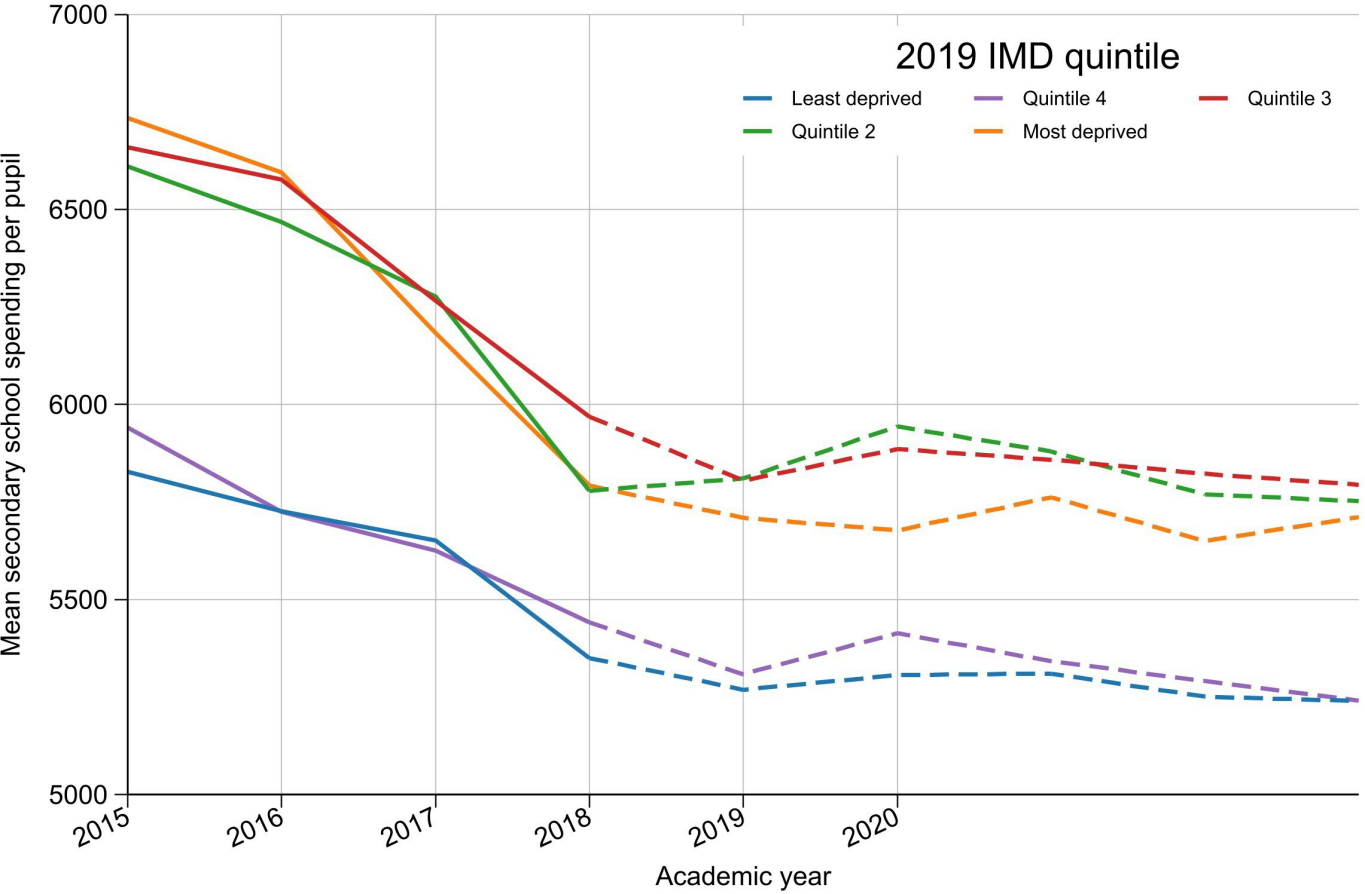
Mean secondary school spending per pupil, by LA deprivation quintile, 2015–2023 (2015/2016 prices). Dashed lines depict the post-NFF period. IMD, Index of Multiple Deprivation; LA, local authority; NFF, national funding formula.

Between 2015 and 2023, mean secondary school spending per pupil decreased by £844 (13%) in real terms, from £6397 in 2015 to £5553 in 2023 (figure 1; online supplemental table S1). This reduction was not felt equally; spending by schools in the most deprived LAs decreased by £1023 per pupil, compared with £588 per pupil in the least deprived quintile. The difference in spending between the most and least deprived LAs has narrowed over time, with more annual average real-term cuts in the most deprived LAs (2%), compared with least deprived LAs (1.7%). Following periods of sustained declines in per pupil school spending in the pre-NFF period (2015–2018), spending was relatively flat in the post-NFF period (figure 1).

**Figure 2:**
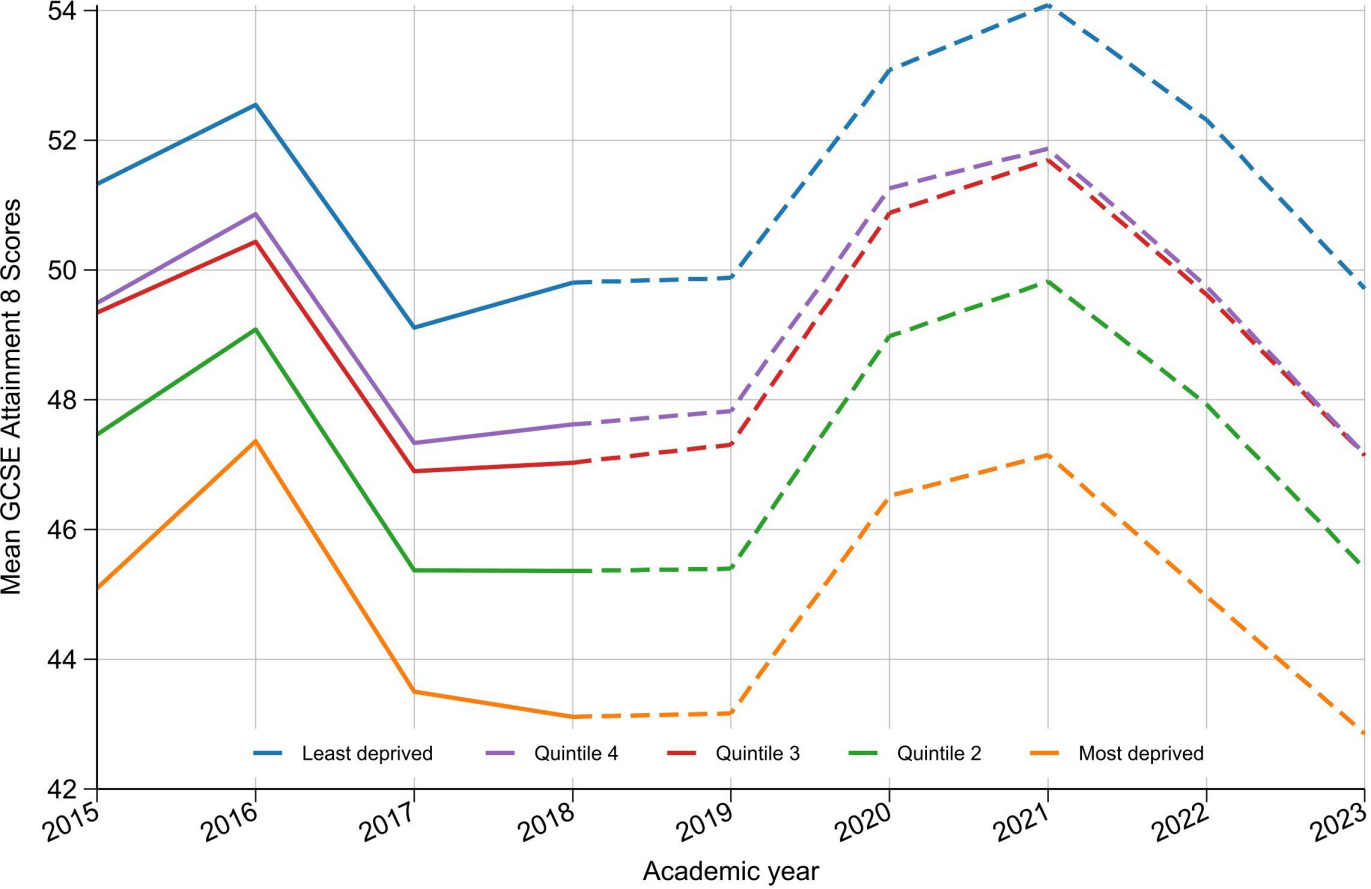
Mean GCSE Attainment 8 scores, by LA deprivation quintile, 2015–2023 (2015/2016 prices). Dashed lines depict the post-NFF period. GCSE, General Certificate of Secondary Education; LA, local authority; NFF, national funding formula.

**Figure 3:**
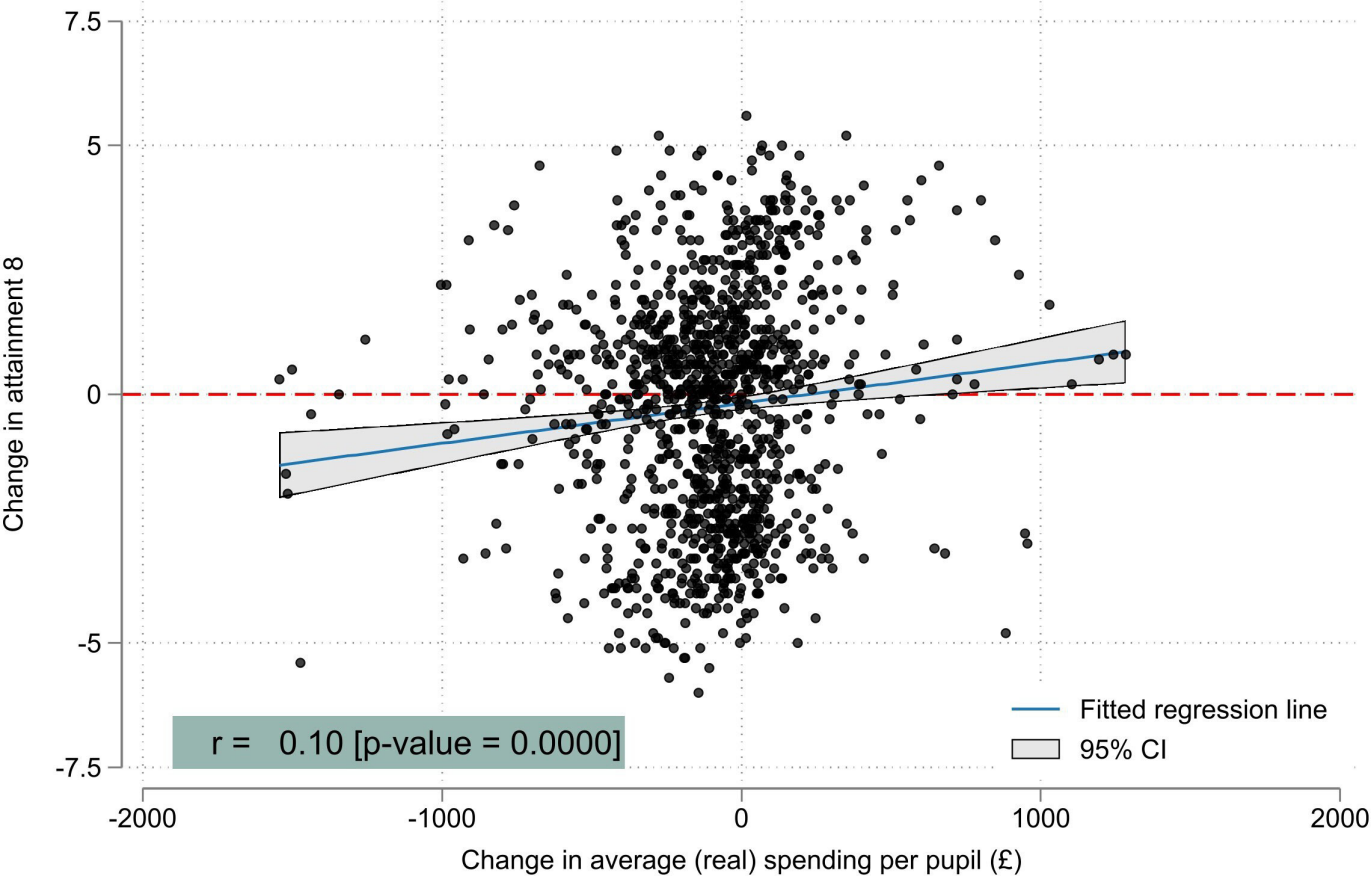
Correlation between change in per pupil school spending and GCSE Attainment 8 scores between 2016 and 2023. GCSE, General Certificate of Secondary Education.

Average annual Attainment 8 scores declined by 4% over the period, from 48.5 mean GCSE Attainment 8 score in 2015 to 46.4 in 2023, and a 2.1 index point difference (figure 2). The pattern in attainment scores over time is similar for all deprivation quintiles with a clear socioeconomic gradient in attainment scores. For all quintiles, attainment is worse now than it was in 2015. The exploratory scatter plots presented in figure 3 showed a weak positive correlation between changes in real term per pupil secondary school spending and changes in GCSE Attainment 8 scores across LAs over the study period (r=0.11; p=0.0001).

### Association between changes in school spending and changes in GCSE attainment

Table 1 shows the fixed-effects components of our model estimating the association between real-term secondary school spending on Attainment 8 scores. Between 2015 and 2023, we estimated that a 10% change in inflation-adjusted school spending per pupil led to a change in Attainment 8 scores by 0.42 index points (95% CI 0.19 to 0.66). In context, a cut to average school spending of £581 (10% of the £5809 average between 2015 and 2023) would decrease mean Attainment 8 scores from 48.3 to 47.9.

**Table 1:** Association between school spending and GCSE attainment across England LAs, 2015–2023

	Relative change in GCSE attainment (95% CI)	Wald test (P value)
Annual school spending (10% change, 2015/2016 prices)	0.42 (0.19 to 0.66)	
IMD quintile		
Q5—most deprived	0.54 (0.17 to 0.92)	
Q4	0.37 (0.04 to 0.70)	
Q3	0.68 (0.22 to 1.14)	
Q2	0.54 (−0.05 to 1.13)	
Q1—least deprived	−0.04 (−0.46 to 0.38)	
Difference in association: pre-NFF and post-NFF		0.22 (0.639)
Before NFF (2015–2018)	0.46 (0.20 to 0.71)	
After NFF (2019–2023)	0.41 (0.15 to 0.66)	
Mundlak χ^2^ (p value)	82.0 (0.000)	

All models adjust for number of teachers as well as proportion of pupils eligible for free school meals, English as an additional language and special educational needs. 95% CIs are based on robust SEs.

GCSE, General Certificate of Secondary Education; IMD, Index of Multiple Deprivation; LA, local authority; NFF, national funding formula.

There was an effect modification using the categorical LA deprivation quintile, with estimated effects varied by deprivation quintile. There was a larger effect in the most deprived LAs (0.54; 95% CI 0.17 to 0.92) compared with the least deprived quintile (−0.04; 95% CI −0.46 to 0.38), but no clear gradient. Also, the association after the NFF policy is not different from the effects in the pre-NFF period (Wald χ^2^=0.22; p=0.639; table 1). The average annual change in GCSE Attainment 8 scores for periods between 2015 and 2018, prior to the NFF, was 0.46 (95% CI 0.20 to 0.71), and not significantly different from the post-NFF estimated association of 0.41 (95% CI 0.15 to 0.66). The Mundlak specification test result is significant (χ^2^=82.0; p=0.000) and supports the estimated model, which accounts for time-invariant unobserved heterogeneity. The sensitivity analysis using data sample excluding periods impacted by the pandemic (2020–2023) further confirmed the significant impact of funding cuts on GCSE attainment (online supplemental table S3). The impact is also higher for LAs in the most deprived quintile. Also, the positive association between school spending and GCSE attainment observed in the baseline models (table 1) is similar after the inclusion of year fixed effects (online supplemental table S5). Lastly, excluding any of the potential confounders had only a small impact on the main point estimates (online supplemental table S4).

## Discussion

Our analysis of the impact of school funding cuts on educational attainment finds that cuts to real-term spending were associated with declining GCSE attainment at the end of secondary education from 2015 to 2023 in England. Our findings show some evidence that the effects of school cuts on educational attainment may have more impact in schools in the most deprived areas. National policy to address inequities in school funding has slightly narrowed the gap in spending between the most and least deprived LAs but has not impacted inequities in educational attainment.

Our analysis adds to prior quantitative evidence assessing the association between increasing school resources and improved student outcomes. An early US study found no evidence of spending on performance. Hanushek^35^ concludes that providing more funding or adopting a different distribution of funding is unlikely to improve student achievement. However, this finding has been challenged in subsequent literature that employed methods that isolated the impact of school funding from family background and other factors.^5 16^ Employing causal inference techniques, including event study and instrumental variable models, Jackson *et al*^36^ investigated the impacts of reform-induced changes in US school finance, demonstrating that increased spending led to measurable improvements in school quality. Also, Pugh *et al*^37^ found a small but significant effect of increasing school expenditure on relative school performance in Australia using a dynamic panel estimator.

Our analysis shows that the difference in spending between the most and least deprived LAs has narrowed over time, with greater cuts to spending in the more disadvantaged areas in the period prior to the implementation of the NFF. Post-NFF spending is relatively stable across all quintiles of deprivation. The most deprived areas continue to receive more funding than more affluent areas, but this is not redressing inequity in student attainment. This may be due to the pre-existing historical structural inequalities in secondary school funding prior to the policy.^38^ This implies that further consideration could be given to modifying the NFF policy to allow for greater allocation of school funding to areas of deprivation, recognising the equity impact that resources could have on those in greatest need who may not have the family and environmental support of their more affluent peers. Further research is needed to understand how best to use school funds to address both inequity in educational attainment and concerning declines in attainment overall.

There are clear policy and practice implications. The greater impact of school cuts on educational attainment in deprived areas implies that funding for schools in these areas should be further considered in relation to need and equity, particularly if we are to address the widening inequalities in attainment in England, which are now the highest in a decade.^39^ Increasing funding in disadvantaged areas and targeted support for deprived schools could provide a mechanism for reducing the stark geographical inequalities across LAs in England.^3^ Investment, together with improved understanding of the enabling and contextual factors in schools, may all affect student experience and attainment. Student attainment is a measurable result of schooling, such as test scores, qualifications, attendance and progression, while student educational experience relates to teaching, the quality of daily life and learning environment in school, well-being, participation, the community and outreach.^13 40^ Adequacy of learning resources, teaching quality and retention are among the critical factors linking school funding to student attainment and well-being.^2^ Protected budgets and funding for student mental health support services, and extracurricular and enrichment activities, together with the integration of well-being indicators into educational performance monitoring, may all help reduce inequalities in student attainment.^41^

One limitation of our study is that we could not directly investigate mechanisms through which the school spending affected GCSE performance, especially the causal impacts of NFF policy intervention in the distribution of school funding in England. However, our estimation approach using LA-level-aggregated school spending and attainment data provides insights to why money and resources matter in specific contexts.^36^ Another factor is the timing of the NFF implementation. There is a need for a longer follow-up analysis to further understand the impact of the NFF implementation. Also, contemporaneous shocks, such as the COVID-19 pandemic, may affect the association between school funding and attainment independently of the NFF.^42 43^ It is important to account for such shocks in our empirical strategy. Our inclusion of period fixed effects does not completely absorb the effects of such shocks, especially since they could have differential impacts across LAs. Our sensitivity analysis excluding the COVID-19 pandemic period shows consistent results, suggesting our findings on the association between school funding and GCSE attainment are not driven by COVID-related shocks.

Additionally, ecological analyses risk introducing bias as measuring spending and outcomes in the aggregate could obscure the differential effects of spending on student attainment, particularly the effects on socially disadvantaged pupils and marginalised groups within and across each LA. However, our analysis does assess the differential impacts of spending on school performance across quintiles of deprivation, indicating larger impacts in the most deprived areas.

## Conclusion

In this analysis, we assessed the impact of school spending in England on educational attainment, whether the association varied by deprivation, and the implications of a national policy related to school funding on the spending and attainment nexus. We found that decreasing school spending due to funding cuts was associated with a decline in GCSE attainment. There was some evidence of a larger impact in the most deprived areas compared with the least deprived areas, and more deprived LAs had greater absolute cut in funding and drop in GCSE attainment. Also, the NFF policy did not impact the strength of the association between spending and GCSE attainment. The current UK government has emphasised the importance of measuring the long-term impacts of their policies and reforms through attainment at the end of secondary school.^7^ This calls for the urgent need for a more equitable approach to allocating and distributing funding across schools, with the potential to improve student performance and reduce geographical inequalities in educational attainment.

## Supplementary material

10.1136/jech-2025-224132 online supplemental file 1

## Data Availability

No data are available.
