# Supplementary material for: School funding and educational attainment in adolescence: a longitudinal ecological study of English local authorities from 2015 to 2023
Source: J Epidemiol Community Health. 2026 Jun 11;80(8):e224132. doi: 10.1136/jech-2025-224132 (PMC13479265; doi:10.1136/jech-2025-224132)
Supplement: Supplementary file 1 [file jech-80-8-s001.docx]

**SUPPLEMENTARY APPENDIX**

Figure S1: Timeline of major schools funding reforms in England (1980 – 2023)

Text Box 1: Structure of Education and State Schools Funding in England.

Education in England and across the United Kingdom (UK) is structured into five stages; early years (or pre-school), primary, secondary, further education and higher education. Section 7 of the UK Education Act 1996, which governs compulsory education, mandates parents to ensure their children of compulsory school age receive adequate education, which must be full-time, efficient and suitable [1]. The compulsory school age commences from when a child is 5 years, and usually their start of primary school, until the end of school leaving age, when they are aged 16, and usually the end of secondary education [2]. The mandatory education can be received from state-funded schools or private, independently-run, education institutions (including home-schooling).

State-funded schools in England providing primary and secondary education to pupils aged 5 to 16 broadly fall into two categories: Local Authority (LA) - maintained schools and academies, which run by non-profit academy trusts. State schools in England are also broadly classified into specialised and mainstream. Special schools are designed to specially provide education for pupils with Special Education Needs (SEN), as set out in the Education Act 1996. Mainstream schools are non-special schools that may provide education for SEN and non-SEN pupils. They can be state-funded or run independently [3].

LA-maintained schools, academies and specialised schools provide tuition-free education, and they are mainly funded through public resources by the DfE, while private (or independent) schools do not receive public funding but raise their funds mainly through student payments and fees. Government funding to schools is provided through two major channels – revenue funding and capital funding. *Capital funding* is usually administered as a distinct allocation by the DfE to improve existing school estates and fund new school buildings, among other functions. *Revenue funding* is used by schools to finance their daily expenditure, including on teacher and support staff salaries, premise maintenance and improvements, grounds maintenance, energy bills, learning resources and teaching materials. Schools have discretion over how they use their revenue funding, which is typically received through government grants.

There have been series of government policies and interventions over the past five decades to reform the school funding system in England (see Figure S1). The most recent of these reforms was the government introduction of the NFF policy, commencing with consultations in 2015/16. The NFF policy was to eliminate the role of local authorities in school funding allocation and provide more equitable funding to schools. It aimed to bridge the funding gap for schools with large numbers of pupils from disadvantaged backgrounds and help raise the attainment of children who perform less well than their more advantaged peers [4].

Prior to the implementation of the NFF policy in 2018/19, the per pupil funding rate received by local authorities was historically defined by central government through hypothecated grant allocation to local authorities and determined by what had been allocated in previous years. These grants were distributed to local authorities and academy trusts through the Dedicated Schools Grant (DSG) which comprised of 3 blocks: the schools block, the high needs and early years block, and the education services grant [5]. Local authorities thereafter allocated funding to schools using locally determined formula. The main school level characteristic factored into local authorities’ devised formulae was the number of pupils, with no explicit consideration of school level characteristics or pupil needs [6,7]. LA-maintained schools received this funding from local authorities, while academies received it directly from independent academy trusts.

Since the implementation of the NFF, the role of local authorities in devising sharing formulas for distributing school funding has gradually been eliminated. The complete elimination of local authorities’ role is known as the hard or direct NFF, whereby central government set a formula applicable across all state-funded schools [4,7]. The alternative to the hard NFF, which has been implemented across most local authorities, is the soft or indirect NFF, which allows for variations in local funding formulae in each LA.

Schools additionally generate their own funding. State-funded schools raise income through donations and private funds, by letting school facilities and services, and voluntary contributions towards, for example, schools’ trips.

**Table S1: Descriptive stats (N=146 Local Authorities)**

|  | **2015** | **2023** | **Absolute, [and %] change from 2015** | **2015 – 2023 Average** |
| --- | --- | --- | --- | --- |
| Spending per pupil (2015 prices) | 6367.2 | 5555.9 | 811.4 [-13%] | 5809.1 |
|  |  |  |  |  |
| IMD quintile |  |  |  |  |
| *Most deprived quintile* | 6734.3 | 5711.4 | 1022.9 [-15%] | 5979.2 |
| *Quintile 2* | 6609.6 | 5752.0 | 857.7 [-13%] | 6031.9 |
| *Quintile 3* | 6658.8 | 5793.8 | 864.9 [-13%] | 6070.4 |
| *Quintile 4* | 5941.1 | 5240.4 | 700.8 [-12%] | 5480.8 |
| *Least deprived quintile* | 5828.0 | 5239.7 | 588.3 [-10%] | 5436.8 |
|  |  |  |  |  |
| GCSE attainment 8 scores | 48.5 | 46.4 | 2.1 [-4%] | 48.3 |
| IMD quintile |  |  |  |  |
| *Most deprived quintile* | 45.1 | 42.9 | 2.2 [-5%] | 44.9 |
| *Quintile 2* | 47.5 | 45.4 | 2.1 [-4%] | 47.2 |
| *Quintile 3* | 49.3 | 47.1 | 2.2 [-4%] | 48.9 |
| *Quintile 4* | 49.5 | 47.2 | 2.3 [-5%] | 49.2 |
| *Least deprived quintile* | 51.3 | 49.7 | 1.6 [-3%] | 51.3 |

**Model specification**

The estimated correlated random-effect model is specified as:

where $att8_{it}$is the GCSE performance measured as the average attainment 8 scores for each local authority $\left(i\right)$ in period $\left(t\right)$. $lrexp_{it}$ is the natural logarithm of school expenditure in 2015 real prices. $Z_{it}$is the vector of covariates including number of teachers, number of full-time equivalent pupils, proportions of pupils eligible for free school meals, English as additional language, and special education needs. $\upsilon_{i}+u_{it}$ is the error component, and are assumed to be independent, with zero means and mutually uncorrelated. $IMD_{i}$ denotes an indicator variable of area deprivation which does not vary with time. IMD is based on the 2019 index of multiple deprivation quintiles. $NFF_{t}$ is an indicator variable for the national funding formula policy and it takes 0 value for periods before the policy (2015 – 2018) and one for periods after NFF (2019 – 2023). The cluster means of the time-varying explanatory variables and their interactions (variables with bars) are included in the model to control for endogeneity.

Our main interest is $\beta_{1}$, the coefficient of school spending which changes across local authorities and time. While IMD and NFF were included and interacted with $lrexp_{it}$ to estimate the average difference in spending impact on GCSE performance across geographical deprivation, and before and after the NFF policy introduction. $\alpha_{t}$ captures the unrestricted period-fixed effects capturing changes in GCSE performance over time. Because of the colinear relationship between the NFF variable, which is just another a period dummy, it difficult to simultaneously estimate the effect of NFF introduction and include year fixed effects in the model. Hence, we considered period fixed effects using coarsened biyearly periods to capture broader trends or unobserved common time trends (Table S2). The underlying rationale for this is that such unobserved common time trends could span multiple years, rather than year-to-year fluctuations.

**Table S2: Coarsened biyearly periods to capture fixed-effects**

| **Academic year** | **Coarsened bi-yearly period** |
| --- | --- |
| 2014/15 | 2014 |
| 2015/16 – 2016/17 | 2016 |
| 2017/18 – 2018/19 | 2018 |
| 2019/20 – 2020/21 | 2020 |
| 2021/22 – 2022/23 | 2022 |

**Table S3: Association between school spending and GCSE attainment across England LAs, before Covid-19 pandemic (2015–2019).**

|  | **Relative change in GCSE attainment [95% CI]** | **Wald test (p-value)** |
| --- | --- | --- |
| Annual school spending  *(10% change, 2015/16 prices)* | 0.93 [0.53, 1.33] |  |
| IMD quintile |  |  |
| *Q5 – Most deprived* | 1.39 [0.98,1.80] |  |
| *Q4* | 0.9 [0.51,1.29] |  |
| *Q3* | 1.46 [0.86,2.06] |  |
| *Q2* | 0.45 [-0.79,1.69] |  |
| *Q1 – Least deprived* | 0.38 [-0.16,0.92] |  |
|  |  |  |
| Difference in association: pre- & post-NFF |  | 2.41 (0.121) |
| *Before NFF (2015 – 2018)* | 0.98 [0.58,1.37] |  |
| *After NFF (2019)* | 0.79 [0.34,1.23] |  |
|  |  |  |
| Mundlak $\chi^{2}$ (p-value) | 94.9 (0.000) |  |

Note: all models adjust for number of teachers, and proportions of pupils eligible for free school meals, English as an additional language and special education needs. 95% CIs are provided in squared brackets and are based on robust standard errors.

The differences in estimates summarised in Table S3 (covering 2015-2019) and the main estimates in Table 1 (2015 – 2023) is explained by the differences in the panel means included in the regressions. The CRE estimator controls for potential endogeneity and correct bias by including in the regression estimation, the panel means of the main regressor $\left(\overline{lrexp_{i}}\right)$, interactions $\overline{\left(lrexp_{it}\times IMD_{i}\times NFF_{t}\right)}$ and covariates $\left(\overline{Z_{i}}\right)$. Importantly, the estimated magnitudes are marginal effects, and they are derived from models with different interaction terms representing conditional associations, rather than single regression coefficients. These magnitudes are conditional on the differences in the model specifications, sample periods covered and their distribution. The differences in the estimated magnitudes indicate that the effect sizes of the changes in average GCSE attainment associated with average per-pupil school spending across local authorities are sensitive to different model specifications.

**Table S4: Associations between school spending by local authority areas and GCSE attainment, with alternative confounder adjustments**

|  | **Relative change in GCSE attainment**  **[95% CI]** |
| --- | --- |
| Unadjusted | 0.24 [-0.11, 0.58] |
| *Excluding:* |  |
| *Number of teachers* | 0.71 [0.31, 1.10] |
| *Free school meals (%)* | 0.69 [0.29, 1.09] |
| *English as additional language (%)* | 0.70 [0.31, 1.10] |
| *Special education needs (%)* | 0.70 [0.31, 1.10] |
| *Adjusted for:* |  |
| *Number of teachers* | 0.24 [-0.10, 0.58] |
| *Free school meals (%)* | 0.68 [0.27, 1.09] |
| *English as additional language (%)* | 0.67 [0.26, 1.09] |
| *Special education needs (%)* | 0.68 [0.26, 1.09] |

**Table S5: Association between school spending and GCSE attainment across England LAs, including year fixed effects (full sample, before and during NFF).**

| **Relative change in GCSE attainment and 95% CI** | **Full sample**  **(2015 – 2023)** | **Before NFF**  **(2015 - 2018)** | **During NFF**  **(2019 – 2023)** |
| --- | --- | --- | --- |
| Annual school spending  *(10% change, 2015/16 prices)* | 0.37 [0.13, 0.61] | 1.15 [0.56, 1.73] | -0.16 [-0.46, 0.13] |
| IMD quintile |  |  |  |
| *Q5 – Most deprived* | 0.69 [0.33, 1.06] | 1.85 [1.25, 2.46] | -0.23 [-0.62, 0.15] |
| *Q4* | 0.51 [0.17, 0.84] | 1.17 [0.62, 1.72] | -0.32 [-1.04, 0.40] |
| *Q3* | 0.42 [0.05, 0.79] | 1.66 [0.77, 2.56] | -0.33 [-0.93, 0.26] |
| *Q2* | 0.3 [-0.24, 0.84] | 0.49 [-1.06, 2.05] | 0.15 [-0.41, 0.71] |
| *Q1 – Least deprived* | -0.10 [-0.52, 0.31] | 0.48 [-0.45, 1.40] | -0.05 [-1.01, 0.91] |
|  |  |  |  |
| Mundlak $\chi^{2}$ (p-value) | 76.58 (0.000) | 29.05 (0.001) | 100.81 (0.000) |

Note: all models include year fixed effects and adjust for number of teachers, and proportions of pupils eligible for free school meals, English as an additional language and special education needs. 95% CIs are provided in squared brackets and are based on robust standard errors.

**References**

[1]	S. Machin, A. Vignoles, What’s the Good of Education? : The Economics of Education in the UK, (2018) 1–100. https://www.torrossa.com/en/resources/an/5580303 (accessed June 2, 2025).

[2]	UK Government, School admissions, GOV.UK. (2025). https://www.gov.uk/schools-admissions (accessed June 2, 2025).

[3]	Education Endownment Foundation, Special Educational Needs in Mainstream Schools, 2025. https://educationendowmentfoundation.org.uk/education-evidence/guidance-reports/send (accessed June 3, 2025).

[4]	Department for Education, National Funding Formula reforms - Schools Bill Factsheet, (2022). https://assets.publishing.service.gov.uk/media/628bab80d3bf7f1f487b7489/National_Funding_Formula_reforms_-_Schools_Bill_Factsheet.pdf (accessed December 6, 2023).

[5]	UK Government, Dedicated schools grant (DSG): 2016 to 2017, GOV.UK. (2015). https://www.gov.uk/government/publications/dedicated-schools-grant-dsg-2016-to-2017 (accessed July 6, 2024).

[6]	Department for Education, School revenue funding Current funding arrangements, 2016. https://consult.education.gov.uk/funding-policy-unit/schools-national-funding-formula/supporting_documents/Current_funding_system.pdf.

[7]	C. Belfield, L. Sibieta, Long-run trends in school spending in England, IFS Report, 2016.
